# Examining variations in perceived barriers and self-efficacy for physical activity among adults in underserved communities

**DOI:** 10.1007/s10865-025-00627-1

**Published:** 2026-01-19

**Authors:** Anqi Deng, Nicole Zarrett

**Affiliations:** 1 Department of Health, Human Performance, & Recreation, University of Arkansas, 308R HPER Building, Fayetteville, AR 72701 USA; 2 Department of Psychology, University of South Carolina, Columbia, SC USA

**Keywords:** School health, Moderate-to-vigorous physical activity, Adults, Underresourced community, Motivation

## Abstract

The purpose of this study was to examine the relations between barriers, self-efficacy, and daily moderate-to-vigorous physical activity (MVPA) among adults within underresourced communities using 7-day accelerometry wear. A total of 84 adult staff from 24 underresourced afterschool programs (ASPs) completed the Self-Efficacy for PA Scale (perceived barriers) and Self-Efficacy for Exercise Questionnaire (self-efficacy). The results indicated no differences in the predominant types of PA barriers by race for adults, but European American adults reported slightly more PA barriers than African American adults within these underrecourced communities. Perceived barriers were negatively related to daily MVPA. Self-efficacy (the mediator variable) was significantly and positively related with daily MVPA. Contrary to what was expected, perceived barriers were positively related with self-efficacy. In the full mediation model, self-efficacy served as a significant mediator between barriers of PA on staff MVPA. This study highlights the negative impact of barriers on ASP staff MVPA that can be attenuated by self-efficacy and suggests that addressing barriers of culture and environmental factors, promoting self-efficacy, and exploring effective model characteristics continues to be an important research direction for future ASP staff health initiatives.

*Trial registration* Connect Through PLAY: A Staff-based Physical Activity Intervention for Middle School Youth (Connect). https://clinicaltrials.gov/ct2/show/NCT03732144. Registered 11/06/2018. Registration number: NCT03732144

## Introduction

Regular physical activity (PA) is essential for promoting and maintaining overall health and well-being (Whitsel et al., 2024). It is associated with a reduced risk of numerous chronic conditions, including cardiovascular disease, hypertension, type 2 diabetes, certain cancers (e.g., breast and colon), and mental health concerns such as depression, anxiety, and stress (Statistics, 2017; Whitsel et al., 2024). Despite the many benefits of PA, the majority (53%) of US adults in the United States are not achieving the prescribed weekly 150 min of moderate to vigorous PA, with only 24% of Americans meeting the PA guidelines for both strength training and aerobic activities (Statistics, 2017; Whitsel et al., 2024). These disparities are even more pronounced among adults from underserved communities, including those from minoritized racial/ethnic groups and low-income populations (Bantham et al., 2021). Structural and systemic barriers, stemming from historical and ongoing policies that privilege European American communities, compound environmental, social, and personal challenges that limit PA access and participation. These may include limited transportation options, unsafe neighborhoods, financial constraints, and a lack of accessible PA resources (Bantham et al., 2021). These structural inequities contribute to reduced PA self-efficacy, which has been shown to negatively influence PA behavior (Rhodes et al., 2020). Although many adults in underresourced communities face significant PA barriers, some remain physically active despite these challenges. This suggests variability in how barriers are experienced and how they influence individual motivation and behavior. However, limited research has examined how different types and frequencies of PA barriers relate to PA self-efficacy and actual MVPA engagement within underserved adult populations.

The current study addresses this gap by exploring PA barriers, self-efficacy, and daily MVPA among adults working in school and afterschool programs (ASPs) located in underresourced communities. ASPs in underserved communities play a critical role by providing safe, structured environments where youth can engage in regular PA, access supportive adult role models, and develop healthy movement habits that may persist into adulthood (Zarrett et al., 2021). ASP frontline staff not only face their own health and wellness challenges but also serve as key influencers of youth PA through their behaviors and engagement. Establishing regular MVPA habits early in life is critical, as youth PA is strongly linked to long-term health trajectories and the likelihood of maintaining active lifestyles into adulthood (Howie et al., 2020). A growing body of research has demonstrated a direct association between staff PA participation and youth PA outcomes in ASP settings. For example, Beets et al. (2015) and Zarrett et al. (2013) found that when staff actively engaged in PA with youth, such as playing games, demonstrating skills, or leading activities, youth exhibited significantly higher MVPA levels. These findings highlight that staff behavior is not only observationally linked to youth PA, but may also drive the motivational climate necessary for sustained youth engagement in PA (Zarrett et al., 2018, 2021). Given these findings, understanding the individual factors that influence ASP staff’s own engagement in PA, particularly perceived barriers and self-efficacy, is essential for developing targeted PA-interventions. Frontline staff who believe in their ability to engage in PA and who perceive fewer barriers are more likely to model and promote PA for youth. Thus, this study takes an important first step toward enhancing the quality and impact of PA programming by identifying the personal challenges and motivations of ASP staff who act as role models and agents of change in the lives of children in underserved communities (Deng et al., 2023, 2024; Zarrett et al., 2021). This knowledge is essential to informing future “healthy school” initiatives that aim to align teacher/staff wellness with broader school- and community-based health promotion goals (Lewallen et al., 2015).

## Perceived barriers to PA

Perceived barriers to PA refer to individuals’ perceptions of the internal and external obstacles that typically prevent them from engaging in PA (Arzu et al., 2006). Internal barriers include beliefs about skill, willpower, abilities, and internal feelings and motivations (Ajzen, 2002; Motl et al., 2005), whereas external barriers include issues concerning access to facilities, neighborhood/community safety (Mendoza-Vasconez et al., 2016), financial constraints (Smith et al., 2017), work responsibility challenges such as multiple jobs, long working hours, lack of time (Bantham et al., 2021), and cultural considerations (Edgerly et al., 2009). A cross sectional study with 1066 adult women and 1036 adult men examined the predictors of the perception of barriers to PA (Herazo-Beltrán et al., 2017). The results showed that among the general population the most common barriers for PA were lack of motivation followed by lack of time. Individuals from low socioeconomic communities were at greatest risk for perceived barriers and these were predominantly in the form of lack of motivation and lack of resources (Herazo-Beltrán et al., 2017). Likewise, adults from different racial/ethnic groups have reported several different individual level PA barriers. In a qualitative study, European American women discussed perceived barriers to PA that were related to the physical environment in rural areas while African American women reported a lack of role models and other social cultural factors such as exposure to socialization messages relaying that exercise was not part of their culture (Wilcox et al., 2005). In another study, time constraints and personal health issues were reported as prominent PA barriers by Latinas, African American, and American Indian adults. In turn, research has evidenced a negative association between perceived barriers and leisure-time PA among national and international adult populations (Herazo-Beltrán et al., 2017; Rosselli et al., 2020; Todorovic et al., 2020).

Although ASP frontline staff are expected to engage in PA with youth as part of the ASP youth development curriculum and mission, underresourced ASPs are typically characterized by a lack of resources/capacity that can interfere with staff engagement with the PA curriculum, including limited funding, lack of facilities and equipment, high staff burnout rates, and minimum enrollment fees (Alliance, 2021; Frazier et al., 2019; Zarrett et al., 2015). In turn, staff report facing greater challenges for promoting underserved youth to be active than other programs with sufficient resources, however, little is known about the barriers staff encounter for their own PA. The aim of the current study is to identify key modifiable factors that account for, at least in part, the degree to which perceived barriers influence daily MVPA among underresourced ASP staff.

### Self-Efficacy as a potential mediator

Social Cognitive Theory (SCT; (Bandura, 2001) has served as a critical theoretical model to inform the promotion of healthy behavior adaptation and disease prevention. SCT describes the interplay between contextual (e.g., perceived barriers, perceived resources, social support), and personal social cognitive factors (e.g., intrinsic motivation, self-efficacy) as a set of determinants that affect the health-related PA beliefs and behaviors of individuals (Bandura, 2001). The self-efficacy component of SCT is considered to be the most powerful and proximal cognitive predictor of behavior (Bandura, 2001), including PA behavior. Self-efficacy represents an individual’s belief that they can successfully undertake a behavior or action required to produce a given outcome (Bandura, 2001). Self-efficacy is theorized to influence the activities that individual’s approach, the effort expended on those activities, and the degree of persistence in the face of failure or adversity (Bandura, 2001). Individuals with higher self-efficacy for a behavior are more likely to initiate and adhere to the behavior and thus, self-efficacy commonly serves as a point of focus for interventions that target a change in behavior (Mo et al., 2011). In particular, considerable evidence has demonstrated a positive association between self-efficacy and PA among adults of various age ranges, race, ethnicity, and gender (Ayotte et al., 2010; Clark & Nothwehr, 1999; McAuley et al., 2006; White et al., 2012). In sum, both theory and empirical studies suggest that self-efficacy is a construct that influences PA behavior and greatly contributes to the spontaneous route of PA behavior change among adults.

According to SCT, behavioral performance, in this case physical activity engagement, is influenced by the interaction between personal, contextual, and social cognitive factors (Schunk & DiBenedetto, 2022). However, there has been limited to no research examining potential variations in the relations between adults’ perceived barriers, self-efficacy, and PA behavior among differing ethnic-racial and socioeconomic communities. National surveys have consistently indicated significant differences in PA across racial groups, with PA among African American adults averaging about 26% lower compared to non-Hispanic European American (Benjamin et al., 2017). Race-related PA differences have been attributed, at least in part, to barriers associated with differences in education, socioeconomic status, time constraints, and residential location (Saffer et al., 2013). Limited evidence also indicates that African American adults report lower general self-efficacy due to socioeconomic factors such as education and income (Assari, 2017). However, there has been only a single study that has examined the mediation role of self-efficacy on the relations between perceived barriers and daily PA (measured by a single survey item) among adults. Findings of this study suggest that self-efficacy mediates the relations between perceived barriers and level of physical activity among student nurses (M_age_ = 24.78 years, SD = 6.88) and health care staff (M_age_ = 41 years, SD = 8.22; (Mo et al., 2011). Although previous studies have evidenced some relations between perceived barriers, self-efficacy, and PA behavior using self-report survey, the relations between these factors using objectively measured MVPA using accelerometers and among the current study’s target population, Black/African American (79.8%) and European American (11.9%) adult staff who live and work in underresourced communities and schools, remains unknown.

Guided by SCT, the purpose of this study was to examine the relations between barriers, self-efficacy and daily MVPA among adults within underresourced communities using 7-day accelerometry wear. To address gaps in previous research, the aims of the proposed study are threefold and include an examination of (a) the amount and types of PA barriers experienced by adults within underresourced communities, (b) whether barriers, self-efficacy, and PA significantly differ within underresourced communities by race, and (c) whether self-efficacy mediates the negative impact of barriers on adult MVPA. It is hypothesized that self-efficacy positively and significantly predicts staff daily MVPA and that perceived barriers negatively and significantly predicts staff self-efficacy and daily MVPA. Based on limited evidence provided by previous studies, it is expected that African American ASP staff perceive greater barriers and lower self-efficacy for PA compared with European American ASP staff. It is also hypothesized that PA self-efficacy significantly mediates the negative relations between perceived barriers and daily MVPA.

## Methods

### Research design and participants

This study is a part of a 5-year randomized clinical trial intervention study (Registration number: NCT03732144). Cross-sectional data were collected from 24 ASPs serving underserved youth using surveys and accelerometers administered to program staff at baseline. The survey data were collected during the ASPs hours (3pm-6pm) with assistance from the research team who had previously received relevant training on how to administer the surveys. ASP-level socioeconomic status was determined by the average free and reduced meal percentages (enrollment includes at least 50% of youth from low-income households, defined by free reduced lunch and minority status). The 24 sites were recruited within the network of Boys and Girls Club community partners of the Midlands in the Southeastern United States during Years 1 through 5 of the trial (September 2018 – June 2024). The ASPs were scattered across small urban, sub-rural, and rural areas. Passive consent procedures were utilized in which staff were informed about the study and the nature of participation and were given the opportunity to opt out of the study if they did not wish to participate in filling out surveys, wearing the accelerometer, or measured for height and weight. This study protocol was approved by the University institutional review board (MASKED). There were 99% of ASP staff within 24 ASPs who met inclusion criteria and consented to participate. A total of 157 ASP staff members were initially recruited at the beginning of the project to participate in the study.

### Variables and measures

Staff were administered surveys at baseline in which they provided their ethnic-racial identity (European American, African American, Asian, Native Hawaiian/Pacific Islander, or Other) and answered questions related to their perceived barriers and self-efficacy for PA. The self-reported survey was administered during ASP hours by the research team and ASP staff had the option of completing it through using an online or paper format.

### Perceived barriers

ASP staff’s total perceived barriers were measured by using the 14-item Self-Efficacy for PA Scale (Sallis et al., 1988). An example item is: “You have a lot of demands at work, is this a current barrier for you?” with a dichotomous response choice of either ‘No (coded as 0)’ or ‘Yes (coded as 1)’. The raw scores, 0 or 1, were aggregated for the calculation of the total perceived barriers scores, with a total maximum perceived barriers cumulative score of 14. In the current study, the range of ASP staff total perceived barriers scores was between 0 and 10. Previous empirical and psychometric studies have indicated adequate concurrent and predictive validity of the Self-Efficacy for PA scale within similar demographic samples (Rogers et al., 2008).

### Self-Efficacy

ASP staff self-efficacy was assessed by the Self-Efficacy for Exercise Questionnaire (Resnick & Jenkins, 2000). The questionnaire consisted of 16 items and a 5-point Likert scale was used to score the participants’ self-efficacy level for each item: 1 = not at all confident; 2 = somewhat confident; 3 = confident; 4 = very confident; 5 = extremely confident. An example item is: “How likely are you to stick to participating in activities that include exercise?” This questionnaire has been widely used across a diverse adult population with strong predictive validity (Wilcox et al., 2005), and the reliability coefficients reported reflect internal consistency, with Cronbach’s alpha values for each questionnaire subscale ranging from 0.972 to 0.975 in the current sample.

### Physical activity

ASP staff’s daily moderate-to-vigorous physical activity (MVPA) was measured using the ActiGraph GT3X accelerometer (ActiGraph LLC), worn on the non-dominant wrist for seven consecutive days. For this study, wear time from 5:00 a.m. to 11:00 p.m. was considered as the full daily wearing period. Data were considered valid if participants recorded a minimum of 480 min (8 h) and a maximum of 600 min of wear time over three or more days (Dillon et al., 2016). This criterion ensures consistency in wear time across participants (Dillon et al., 2016). Since all participants met these wear-time criteria, missing data imputation was not applied. ActiLife software (Version 6.13.3) was used for data download, yielding raw acceleration data. Analysis was conducted in R using the GGIR package with the Euclidean Norm Minus One (ENMO) metric, without imputation (Van Hees et al., 2013). Physical activity intensity thresholds were defined using the Hildebrand et al. (2014) calibration approach, which links accelerometer-derived movement-related acceleration (expressed in milligravity units, mg) to energy expenditure. Based on this method, moderate activity was defined as 201–707 mg and vigorous activity as ≥ 707 mg. Although participants were instructed to wear the device for a full week, more than half of the sample wore the device for only 3–5 days, which limits the precision of weekly PA estimates. Accordingly, daily MVPA values reported in this study are based on valid wear days and then averaged to estimate daily PA.

### Race

ASP staff were asked to provide their race in a self-reported survey. For the current study, participants were self-identified as European American (*n* = 10) and Black or African American (*n* = 67). There were two staff members who identified as Asian (international), and five staff members who identified as other/multiracial who identified as being from racially/ethnic-based minoritized communities. Therefore, we included these 7 staff members as part of the Black/African American sample in the current study to compose a dichotomous variable indicating a participants’ membership in a racially-ethnically minoritized community (*n* = 74).

### Statistical analyses

During data collection, 15 staff members either voluntarily left their positions or were terminated by their employer. Participants with incomplete data included those who provided only survey responses without corresponding accelerometer data meeting the strict requirement of at least 8 h per day for a minimum of three days, as well as those who provided accelerometer data without completing the survey. The high attrition rate among staff in this study can be attributed to these stringent accelerometer data collection criteria, which made adherence challenging. Consequently, data from these 71 staff members were excluded from the final analysis. Chi-square tests using baseline survey data collected on all participants (*n* = 155) indicated that there were no significant differences for gender (*χ*^*2*^ = 1.57, *p* = .21) and race (*χ*^*2*^ = 2.09, *p* = .55) between ASP staff who were included in the data analysis (*n* = 84) and those who were not included (*n* = 71). In addition, results also showed that there were no significant differences for perceived barriers (*χ*^*2*^ = 20.57, *p* = .06), self-efficacy (*χ*^*2*^ = 7.88, *p* = .85) and daily MVPA (*χ*^*2*^ = 65, *p* = .44) between ASP staff who were included in the data analysis and those were not included. For the current study, the final sample included 84 ASP staff, predominantly self-identified as female (75%) and Black/African American (79.8%), who completed both survey and daily MVPA data at the baseline.

**Fig. 1:**
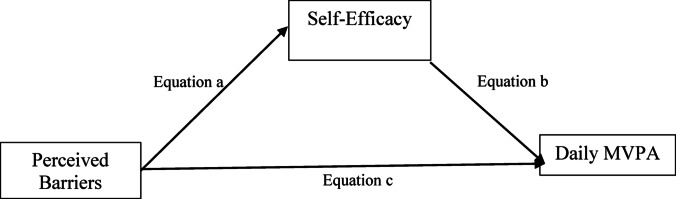
The hypothesized model for research aim 3 (a’ – c’ refers to regression coefficients).

**Fig. 2:**
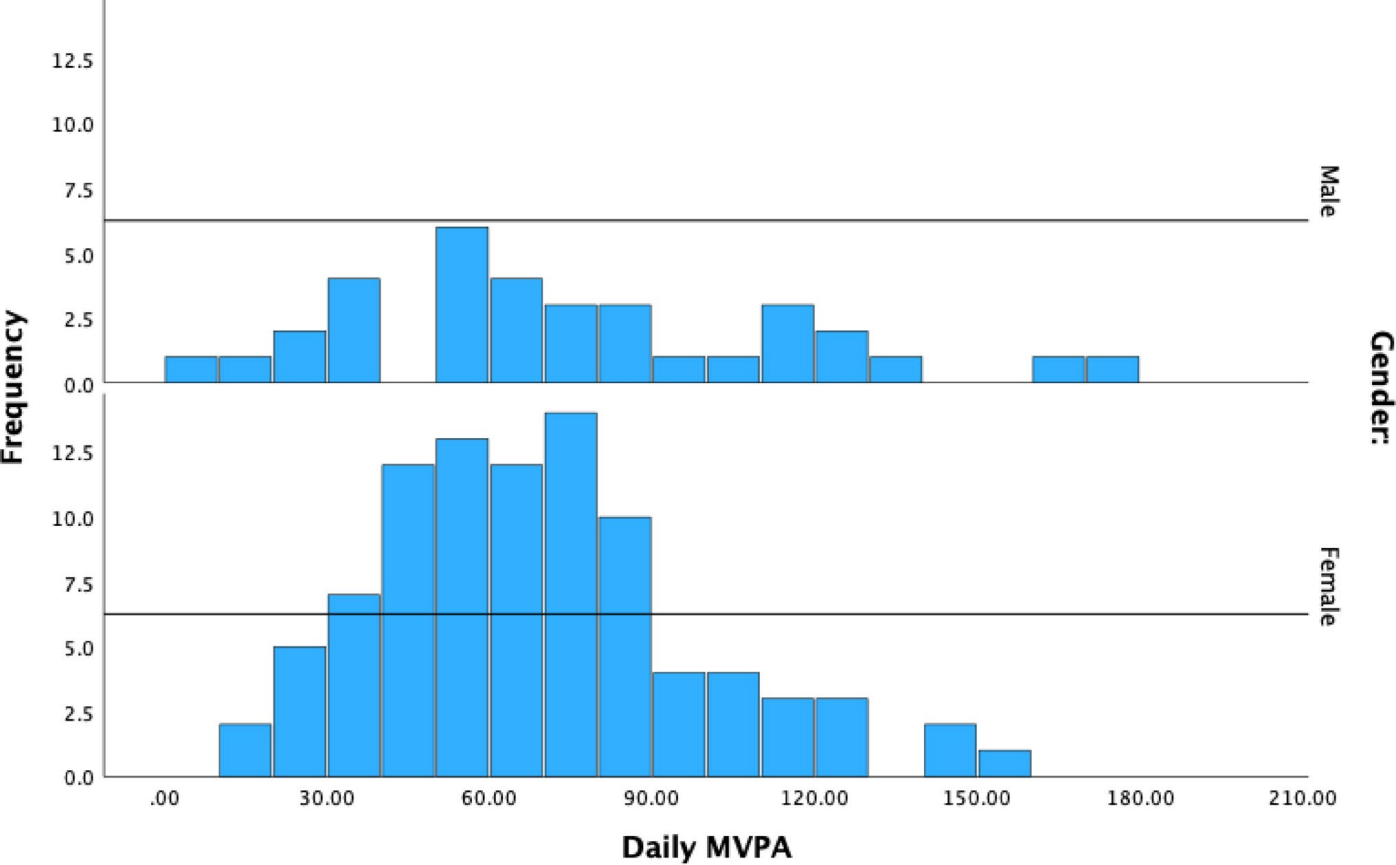
Histogram of Daily MVPA minutes levels by gender

Descriptive analysis was conducted for perceived barriers, self-efficacy and daily MVPA. To address the first research aim, linear regression analyses were conducted to examine differences in staff perceived barriers, self-efficacy, and daily MVPA by race. To address the second research aim, Hayes’ PROCESS v3.5.3 macro analysis was used to test the hypothesized model in Fig. 1. Hayes’ PROCESS macro is a regression analysis modeling tool to assist identifying single or multiple mediators or moderators in associations (Hayes & Scharkow, 2013). Hayes’ PROCESS macro is a suitable choice in which the variables are all directly observed/measured variables as opposed to aggregated latent variables (Hayes & Scharkow, 2013). The advantages of using Hayes’ PROCESS macro are that it generates direct and indirect effects in the mediation models and the maximum likelihood estimation method maximizes the probability of observing the dataset given a model and its parameters (Hayes & Scharkow, 2013). The method includes a bootstrap method; a recommended number of iterations (5,000; (Hayes & Scharkow, 2013) was employed in this analysis. All analyses were conducted using IBM SPSS 29 and a value of *p* < .05 was considered statistically significant. Table 1 reports descriptive statistics of self-efficacy, perceived barriers, and daily MVPA. According to Kline (2011), the skewness indices ranged between − 2 and 2 and the kurtosis indices between − 7 and 7, suggesting that the univariate normality assumption was not violated. The results show that most of ASP staff in this study are moderate-to-highly active adults with an average of 79.42 min MVPA per day (Kline, 2011) (Fig. 2).

**Table 1:** Descriptive results for self-efficacy, perceived barriers, and daily MVPA.

	Race							
	European American				Predominantly African American			
	Mean	SD	Skewness	Kurtosis	Mean	SD	Skewness	Kurtosis
Self-efficacy	3.14	0.38	− 0.30	− 1.44	2.87	0.59	− 0.48	0.38
Perceived barriers	7.40	2.32	0.93	0.02	5.18	3.34	0.47	− 0.46
Daily MVPA	81.94	25.28	0.085	− 0.14	79.08	35.38	0.58	− 0.49

## Results

### Aim 1: types of PA barriers most frequently endorsed

Table 2 reports frequency results for the amount and types of barriers experienced within underresourced communities. Across all participants, the three most endorsed perceived barriers were (a) having limited amounts of time; (b) feeling tired; (c) having a lot of demands at work. Among predominantly African American ASP staff, all 14 types of barriers were endorsed as perceived obstacles to PA participation, and European American staff reported 13 out of the 14 types of barriers, only excluding “feeling bored by the program or activity”. Specifically, among European American ASP staff, the three most endorsed perceived barriers were: (a) having limited amounts of time (100%); (b) having a lot of demands at work (80%); (c) feeling tired (80%). The three least endorsed perceived barriers were (a) having guest staying in home (10%); (b) having to exercise alone (20%); (c) lacking enjoyment in PA and an equivalent number of adults reporting feeling depressed (20%). Among predominantly Black/African American ASP staff, the three most endorsed perceived barriers were (a) having limited amounts of time (58.1%); (b) feeling tired (48.6%); and (c) having a lot of demands at work (44.6%). The three least endorsed perceived barriers among predominantly Black/African American staff were (a) feeling bored by the program or activity (8.1%); (b) having guest staying in home (12.1%); (c) feeling depressed (16.2%).

**Table 2:** Frequency results for amount and types of barriers

	Is this a current barrier for you?		
	European American (*n* = 10)	Predominantly African American (*n* = 74)	Across all participants (*n* = 84)
*Social/Environmental level barriers*			
1. Family demanding more time from you	6(60%)	25(33.8%)	31(36.9%)
2. You have household chores to do	6(60%)	24(32.4%)	30(35.7%)
3. You have guest staying in your home	1(10%)	9(12.1%)	10(11.9%)
4. The weather was bothering you	5(50%)	22(29.7%)	27(32.1%)
5. You have a lot of demands at work	8(80%)	33(44.6%)	41(48.8%)
6. Your friends want to socialize?	2(20%)	13(17.6%)	15(17.9%)
7. You have limited amounts of time	10(100%)	43(58.1%)	53(63.1%)
*Individual level barriers*			
8. You are feeling lazy	7(70%)	35(47.3%)	42(50%)
9. You did not enjoy physical activity	2(20%)	13(17.6%)	15(17.9%)
10. You have to exercise alone	1(10%)	17(23.0%)	18(21.4%)
11. You felt tired	8(80%)	36(48.6%)	44(52.4%)
12. You felt stressed	6(60%)	29(39.2%)	35(41.7%)
13. You felt depressed	2(20%)	12(16.2%)	14(16.7%)
14. You were bored by the program or activity	0(0%)	6(8.1%)	6(7.1%)

### Aim 2: differences in barriers, self-efficacy, and MVPA by race

Linear regression analyses revealed that there were significant differences in perceived barriers (*β* = −  2.22; *p* = .046) by race, but there were no differences by race in daily MVPA (*β* = −  2.86 *p* = .81) and self-efficacy (*β* = − 0.27; *p* = .172). Findings indicate that predominantly African American ASP staff had significantly fewer perceived barriers than European American ASP staff with the total amount of variance explained by 21%.

### Aims 3: self-efficacy as a mediator of barriers on MVPA

**Fig. 3:**
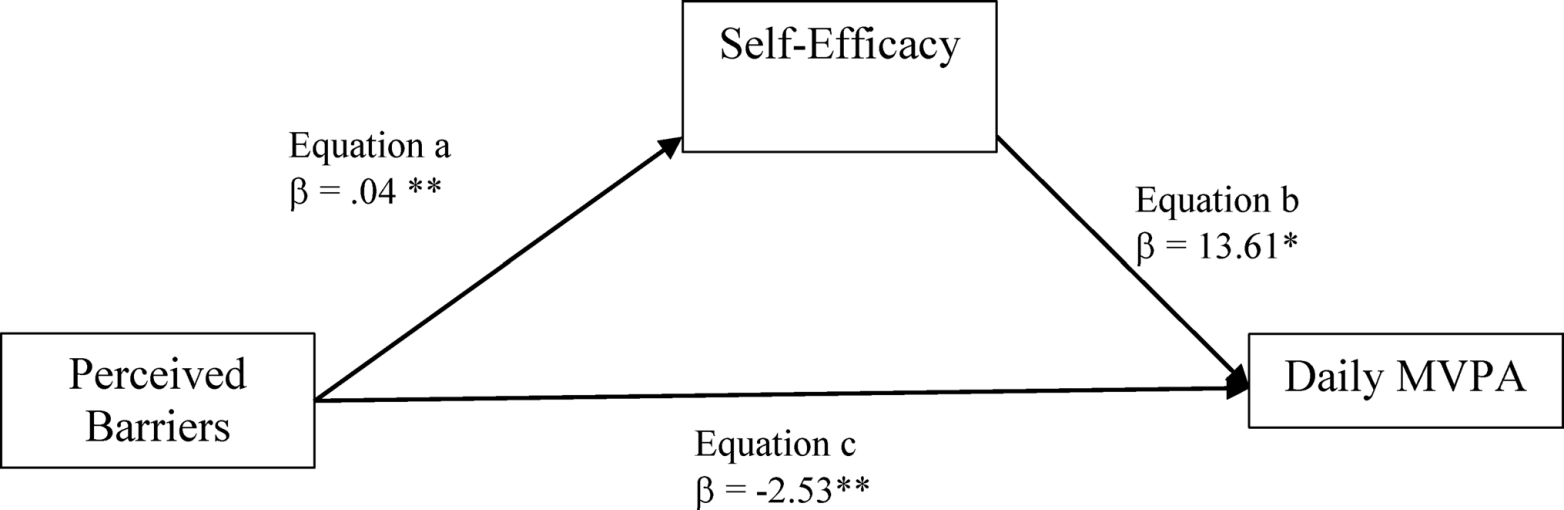
Perceived barriers mediation models of the relations between self-efficacy and daily MVPA among all ASPs staffs

The mediation analysis results shown in Fig. 3 indicates that self-efficacy significantly mediates the relations between perceived barriers and daily MVPA. In the first regression step (equation a), perceived barriers was significantly and positively related with self-efficacy (coefficient = 0.04, *p* = .02). In the second step (equation c), the regression coefficient of perceived barriers on the dependent variable (daily MVPA) was significant and negatively associated (coefficient = −  2.5, *p* = .03). In the last regression model, the mediator variable (self-efficacy) was significantly and positively related with staff daily MVPA (coefficient = 13.62, *p* = .04). Finally, the indirect effect was significant (indirect effect = 0.59; 95% CI = 0.003 to 1.76), indicating self-efficacy was a significant mediator in this model.

## Discussion

This study set out to examine the relations between barriers, self-efficacy, and daily MVPA among adults within underresourced communities using 7-day accelerometry wear. To address gaps in previous research, the aims of the proposed study are threefold and include an examination of (a) the amount and types of barriers experienced within underresourced communities, (b) whether barriers, self-efficacy, and PA significantly differ within underresourced ASPs by race, and (c) whether self-efficacy mediates the negative impact of barriers on adult MVPA. Although previous evidence showed that 26% of male and 35% of female were insufficiently physically active to meet the national recommendation of at least 75–150 min of moderate to vigorous intensity aerobic PA per week (Saint-Maurice et al., 2021), our sample, despite their barriers, reported higher MVPA than other earlier research conducted from a diverse underserved sample, indicating that ASP staff are still participating at adequate levels of daily PA (Bantham et al., 2021). However, there were still 31% of ASP staff who reported daily MVPA less than 60 min and 3.6% of ASP staff who reported minimal daily MVPA (28 –30 min/day) in the current sample. The results showed that (a) having limited amounts of time was reported as the most endorsed social/environmental level barriers among all participants; (b) feeling tired was reported as the most endorsed individual level barriers among all participants. Overall, our findings indicate that European American ASP staff, on average, experienced a greater number of barriers to PA (individuals reported an average of 7.4 [52.8%] out of the total 14 total perceived barriers listed) as compared to the study’s predominantly Black/African American ASP staff (who reported an average 5.18 [37%] out of 14 total perceived barriers). Contrary to what was expected, among this unique sample of ASP staff within underresourced communities there were no differences in self-efficacy or device-measured daily MVPA by race, predominantly African American and European American adults on average had moderate self-efficacy (averaging somewhat confident to confident on the five-point scale) and similar amounts of high daily MVPA. Aligned with previous research and theory, results showed that perceived barriers was negatively related to daily MVPA and self-efficacy (the mediator variable) was significantly and positively related with daily MVPA. Contrary to findings of previous research, perceived barriers were positively related with self-efficacy. As expected, the results of the full mediation model aligned with our hypotheses, with self-efficacy shown to function as a significant mediator between barriers of PA on staff MVPA.

The primary barriers to PA among adults in our study, namely having limited amounts of time, feeling tired, and having a lot of demands at work, align with previous studies that showed a lack of time due to work commitments was the most important “external barrier” (Sharifi et al., 2013), and one of the most common personal barriers was being “tired” (Allison et al., 1999; Dunton & Schneider, 2006; Louw et al., 2012; Sharifi et al., 2013). Previous studies also identified having “lack of motivation” as another primary personal barrier (Allison et al., 1999; Dunton & Schneider, 2006; Louw et al., 2012; Sharifi et al., 2013) which did not align with our sample of ASP Staff. Rather, compared to previous research, our findings also highlighted “feeling stressed (41.7%)” as a unique barrier that was related to working with greater challenges and less resources within underresourced communities. Stress was a particularly salient barrier among this study’s small sample of European American adults (60%). Additionally, the results also indicated that “bored by the program or activity”, “having guest staying at home”, “did not enjoy physical activity/friends want to socialize” as the least common barriers to PA among ASP staff in the underresourced community. The delivery and management of PA programming is one part of an ASP staff job requirement; thus, it may be that staff learn to enjoy PA as part of their daily exposure in the ASP or that the position is particularly attractive to individuals who already enjoy PA and value supporting the health behaviors of youth. Therefore, inactivity among ASP staff is less likely to result from motivational-based individual-level reasons (e.g., lack of motivation and/or dislike of PA) but rather social/environmental factors (time constraints due to other life/work demands) and other physical and mental health-based individual-level reasons (e.g., felt tired/stressed) are more common barriers to their engagement in PA. These findings may have practical and clinical importance for public health and PA interventions among adults in school and other community settings where PA is an expected part of the work setting (Dishman & Sallis, 1994; Sallis et al., 1992).

Previous studies with predominantly European American adults who completed higher education (beyond high school) within more resourced communities have found that self-efficacy was inversely related to the number of perceived barriers (Stutts, 2002). Contrary to our hypotheses and this previous research, our findings showed that among this study’s sample of underserved predominantly Black/African American ASP staff, higher perceived barriers was significantly related to higher self-efficacy. Recently, researchers have argued that influences in the environment and culture can impact individuals’ motivational orientations and perceptions of barriers (Schunk & DiBenedetto, 2022), and thus, principles of Bandura’s theory are context dependent (Schunk & DiBenedetto, 2022). For example, studies showed that individuals in Western cultures (e.g., U.S., Canada) tend to report higher self-efficacy than those in non-Western Cultures (e.g., Japan, China(Klassen, 2004; Klassen & Chiu, 2010). The results of the current study align with this proposed cultural-contextual framework which suggests the importance of considering ways in which the meaning of self-efficacy may be influenced by cultural and environmental factors in today’s diverse societies (Schunk & DiBenedetto, 2022). Despite the study findings providing further evidence of the negative impact that barriers can have on adult moderate to vigorous physical activity (MVPA), among our sample of moderate-to-highly active adults in under-resourced communities, the presence of barriers may have reinforced individuals’ self-efficacy to remain physically active. This is particularly notable given that these adults are in professions where they are expected to engage in PA with youth. The findings of this study provide preliminary evidence for researchers to develop future culturally relevant PA interventions for adults in underresourced communities. Other cultural mechanisms that address staff stress and time constraints may need to be considered. Intervention components that promote positive self-esteem, cultural pride, social resources such as greater engagement with spirituality and social networks and support have been proposed to address ethnic/racial-related stressors and promote minoritized adults’ PA and health-related quality of life (Joseph et al., 2015).

Our findings indicate that underserved, predominantly Black/African American staff on average may have an important set of personal and/or social resources to maintain high efficacy to engage in PA even in the face of more daily life challenges for PA. Overall, individuals within underresourced communities rely on greater intrapersonal resources, like self-efficacy, with increasingly more barriers present within their social-contextual environment, and also learn they are able to expand the effort, motivation and persistence necessary to overcome barriers to regularly engage in PA. As expected, and aligned with previous research (Gillison et al., 2017), positive relations were found between MVPA and self-efficacy beliefs in the current study, and reinforces the importance of fostering PA self-efficacy beliefs for behavior change (Dishman et al., 2019). Our findings suggest that self-efficacy not only positively predicts daily MVPA, it also significantly attenuates the negative impact of barriers on daily MVPA which is beneficial for all staff in our study with both lower and higher daily MVPA. The mediating effects of self-efficacy for overcoming social/environmental and individual-level barriers on staff daily MVPA highlight a promising intervention target. However, because ASP staff may engage in PA as part of their job responsibilities, these findings may not fully generalize to adults in underresourced communities with more sedentary occupations. Future interventions should consider how occupational demands shape PA opportunities and explore whether similar mechanisms apply across diverse work settings.

In summary, this study provides empirical evidence supporting a significant association between self-efficacy and daily MVPA among ASP staff within underresoured ASPs. To the knowledge of the authors, this is the first paper to explore self-efficacy as a mediator to explain the negative impact of barriers on adult MVPA. There are several strengths of this study, including a focus on objectively measured MVPA using accelerometers, focus on ASP staff within underresourced communities, and a diverse sample of participants that included representation from both European American and predominantly Black/African American populations. However, it is important to acknowledge that the study used a relatively small sample size, with a notably small sample of European American adults, and all participants were from the southeastern United States, which limits generalizability. This restricts the generalizability of the findings to broader populations, and future research should aim to include larger, more diverse samples across different geographic regions to enhance the external validity of the results. This study’s cross-sectional design limits the ability to draw conclusions about the directionality or causality of the observed associations. Notably, the use of mediation analyses with cross-sectional data is a limitation, as these analyses presume a temporal ordering among variables that cannot be empirically tested without longitudinal data. As such, interpretations of mediation effects should be viewed as exploratory and interpreted with caution. Future research should examine the proposed model using longitudinal or quasi-experimental designs to better assess causal pathways. Additionally, while participants were instructed to wear the accelerometer for seven consecutive days, over half of the sample provided only 3–5 valid days of data. Although this met our inclusion criteria, the variability in wear time limited our ability to estimate total weekly MVPA with confidence. To minimize bias, we reported average daily MVPA based on valid days; however, this may not fully capture participants’ typical weekly activity. Future studies should aim to improve device adherence through enhanced protocols and support strategies to enable more robust weekly estimates and a clearer picture of physical activity patterns in this population. Finally, qualitative methods, such as in-depth interviews, are needed to capture the complexity of contextual, social, and personal barriers to physical activity among ASP staff and to inform the design of comprehensive, multilevel interventions that are culturally responsive and community-centered.

## Conclusions

ASP staff play a prominent role in delivering PA curriculum and act as key socializers and role models to ensure youth engagement in PA. Findings of this study showed that self-efficacy positively and significantly predicted daily MVPA among all adults, and the mediation effect of self-efficacy was supported. This study highlights the negative impact of barriers on underresourced ASP staff MVPA that can be attenuated by self-efficacy. Participants also reported having limited amounts of time, feeling tired and having a lot of demands at work. as their major barriers for PA. These results will be used to optimize future ASP staff-based health and PA interventions. Hence, the findings of this study suggest that addressing barriers of culture and environmental factors, promoting ASP staff self-efficacy, and exploring other psychosocial factors can be an important research direction for the future ASP staff health initiatives. Additionally, future studies are needed to examine other potential motivation and behavioral factors that can also serve as mediators for attenuating the negative impact of perceived barriers on the daily MVPA of adult staff in underresourced schools and communities.

## Data Availability

A copy of survey instrument questions will be provided upon request to the corresponding author. Participant data are not publicly available due to human subject research protections but qualified academic researchers may send data requests to the corresponding author for review. All use of data would be subject to confidentiality and data-use agreements.
